# AAV2 can replicate its DNA by a rolling hairpin or rolling circle mechanism, depending on the helper virus

**DOI:** 10.1128/jvi.01282-24

**Published:** 2024-10-09

**Authors:** Anouk Lkharrazi, Kurt Tobler, Sara Marti, Anna Bratus-Neuenschwander, Bernd Vogt, Cornel Fraefel

**Affiliations:** 1 Institute of Virology, University of Zurich, Zurich, Switzerland; 2 Functional Genomic Center Zurich, Zurich, Switzerland; International Centre for Genetic Engineering and Biotechnology, Trieste, Italy

**Keywords:** adeno-associated virus, adenoviruses, herpes simplex virus, viral replication, DNA synthesis, DNA polymerase

## Abstract

Adeno-associated virus type 2 (AAV2) is a small, non-pathogenic, helper virus-dependent parvovirus with a single-stranded (ss) DNA genome of approximately 4.7 kb. AAV2 DNA replication requires the presence of a helper virus such as adenovirus type 5 (AdV5) or herpes simplex virus type 1 (HSV-1) and is generally assumed to occur as a strand-displacement rolling hairpin (RHR) mechanism initiated at the AAV2 3′ inverted terminal repeat (ITR). We have recently shown that AAV2 replication supported by HSV-1 leads to the formation of double-stranded head-to-tail concatemers, which provides evidence for a rolling circle replication (RCR) mechanism. We have revisited AAV2 DNA replication and specifically compared the formation of AAV2 replication intermediates in the presence of either HSV-1 or AdV5 as the helper virus. The results confirmed that the AAV2 DNA replication mechanism is helper virus-dependent and follows a strand-displacement RHR mechanism when AdV5 is the helper virus and primarily an RCR mechanism when HSV-1 is the helper virus. We also demonstrate that recombination plays a negligible role in AAV2 genome replication. Interestingly, the formation of high-molecular-weight AAV2 DNA concatemers in the presence of HSV-1 as the helper virus was dependent on an intact HSV-1 DNA polymerase.

**IMPORTANCE:**

AAV is a small helper virus-dependent, non-pathogenic parvovirus. The AAV genome replication mechanism was extensively studied in the presence of AdV as the helper virus and described to proceed using RHR. Surprisingly, HSV-1 co-infection facilitates RCR of the AAV2 DNA. We directly compared AdV5 and HSV-1 supported AAV2 DNA replication and showed that AAV2 can adapt its replication mechanism to the helper virus. A detailed understanding of the AAV replication mechanism expands our knowledge of virus biology and can contribute to increase gene therapy vector production.

## INTRODUCTION

Adeno-associated virus (AAV) is a small, non-pathogenic, helper virus-dependent parvovirus (1). The AAV2 genome consists of a 4.7 kb long, single-stranded (ss) linear DNA with two coding regions, *rep* and *cap*. The coding regions are flanked by inverted terminal repeats (ITR) (2). The AAV ITRs consist of 125 nt of palindromic sequences termed A, A′, B, B′, C, and C′, followed by a 20 nt unique D sequence adjacent to the terminal resolution site (*trs*) (3). Although the ITRs are non-coding, they constitute an important part of the AAV genome, as they self-assemble into a double-hairpin structure that serves as a primer for DNA replication and as a packaging signal (4, 5). Although AAV can infect host cells in the absence of helper factors, it can only replicate if the cellular environment undergoes a dramatic change (6, 7) or in the presence of helper factors provided by specific helper viruses. These helper viruses include adenoviruses (AdV) and herpesviruses (HSV) (8–10) among others. Additionally, cellular stress, such as UV radiation or carcinogens, can induce AAV2 replication to a certain extent (7, 11).

HSV-1 is a large, enveloped virus of approximately 200 nm in diameter with a linear 152 kbp long dsDNA genome (12). The genome is replicated by the HSV-1 polymerase UL30/UL42 and packaged into preformed capsids. Interestingly, viral genomes are only packaged if longer-than-unit-length viral DNA is present (13); therefore, concatemerization of HSV-1 genomes must take place before packaging. In early work, it was suggested that the HSV-1 genome circularizes and enables rolling circle replication (RCR) which inherently leads to concatemers (14). More recent work suggests concatemerization through recombination mediated by ICP8 and UL12 (15, 16).

The minimal helper factors necessary to support AAV replication in AAV and HSV-1 co-infected cells include the HSV-1 helicase primase complex composed of the UL5, UL8, and UL52 proteins and the HSV-1 ssDNA-binding protein ICP8 (17). Although the HSV-1 polymerase (HSV-1 pol), consisting of UL30 and UL42, is not strictly necessary for AAV genome replication, it was shown to enhance the process (18, 19).

AdVs are nonenveloped viruses with a linear, non-segmented dsDNA genome of about 35–36 kbp (20). The AdV genome is replicated by the AdV polymerase through a protein-primed strand-displacement mechanism [reviewed in reference (21)]. The essential helper factors provided by AdV for AAV genome replication include E1A and E2A which are needed for the activation of the AAV *p5* and *p19* promoters (22–24), E1B55K which in concert with E4orf6 promotes second strand synthesis and viral DNA replication (25, 26) and the VA RNA which is required for efficient synthesis of AAV structural proteins (27). Interestingly, in AdV supported AAV genome replication, the cellular polymerase δ was shown to be essential, while the AdV polymerase was not (28, 29).

**Fig 1:**
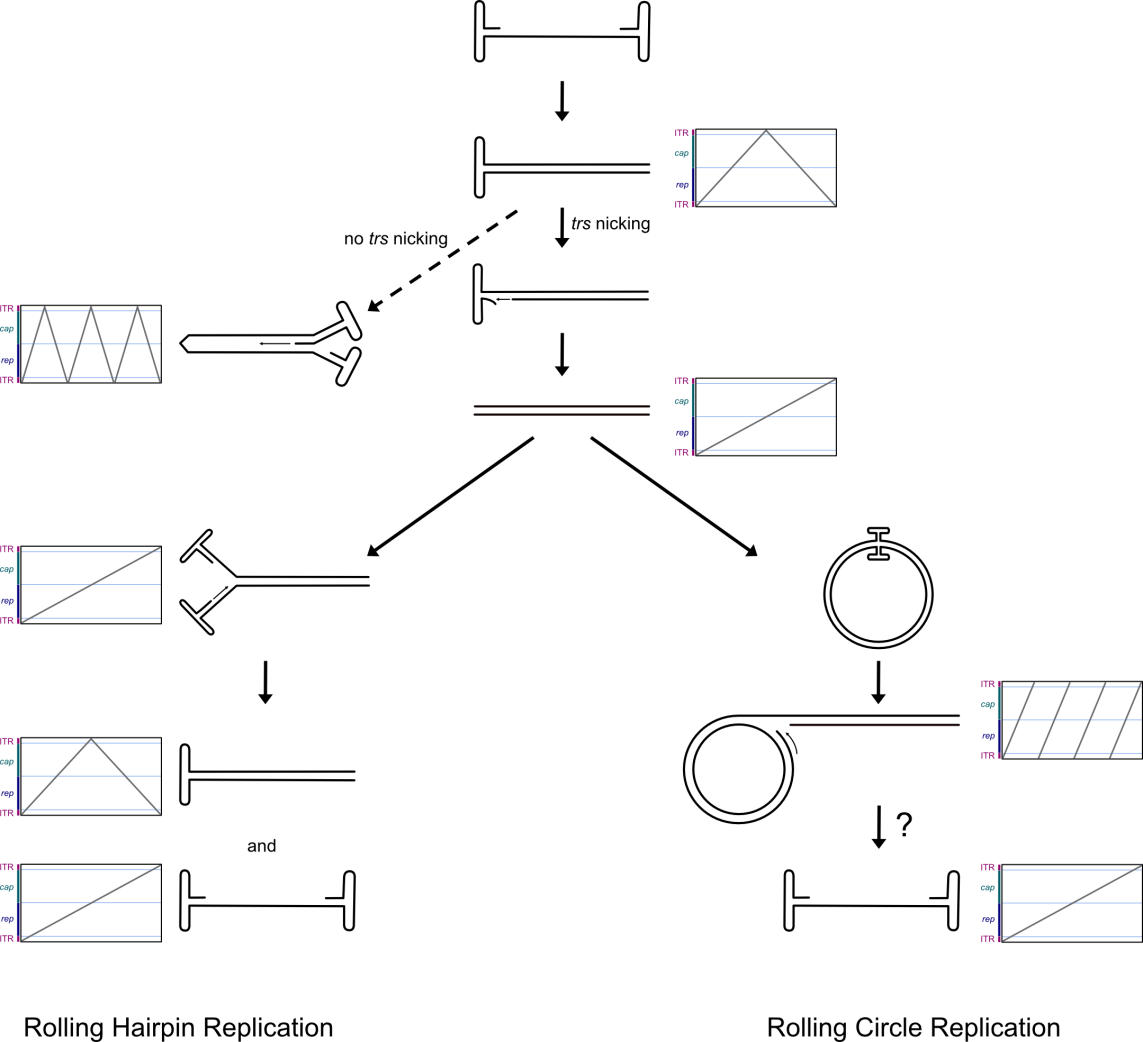
Schematic representation of RHR and RCR AAV2 replication mechanisms. Corresponding dot plots from Oxford Nanopore sequencing are indicated. Circular and single-stranded AAV2 DNA cannot be sequenced due to library preparation and sequencing mechanism.

AdV-supported AAV genome replication is suggested to occur in a rolling hairpin mechanism (RHR, Fig. 1) using the ITR as a primer to initiate second-strand synthesis, which leads to the formation of a covalently closed duplex structure (30). Nicking by Rep68 or Rep78 at the *trs* produces a 3′-end that serves as a primer for the synthesis of a new ITR, resulting in the resolution of the duplex structure (31). In a process termed re-initiation, hairpins form at the ends of the genome and present a new 3′-end primer for DNA synthesis that displaces and releases a single-stranded AAV genome. The newly synthesized AAV DNA is then packaged into preformed capsids (32).

There is ample experimental evidence supporting this model. For example, two distinct ITR orientations, termed ‘flip’ and ‘flop’, have been observed, with their relative positioning found to be independent of each other (2, 33). The ITRs can act as the origin of DNA replication (3, 5, 34). Covalently closed double-stranded (ds) monomer structures as predicted by the RHR model have, indeed, been isolated from AAV-infected cells (35), and site-specific nicking of the *trs* has been observed (31, 36, 37).

Recently, we provided evidence that AAV2 replicates its DNA using an RCR (Fig. 1) rather than an RHR mechanism when HSV-1 is the helper virus (38), suggesting that AAV2 can adapt the genome replication strategy to the helper virus present. Here, we directly compared the AAV2 DNA replication intermediates formed in the presence of either HSV-1 or AdV5. The data are consistent with a preferential RHR in the presence of AdV5 and preferential RCR in the presence of HSV-1. We also demonstrate that recombination plays a negligible role in AAV2 genome replication.

## RESULTS

### Oxford Nanopore sequencing reveals different AAV genome replication intermediates depending on the helper virus, HSV-1 or AdV5

We have recently shown that HSV-1 co-infection facilitates RCR of AAV2 DNA (38), while earlier reports have described AAV to replicate via an RHR mechanism. To investigate this discrepancy, we directly compared AAV2 genome replication when supported by either HSV-1 or AdV5. To analyze AAV2 DNA replication intermediates, we employed Oxford Nanopore sequencing, following the previously described method by Meier et al. (38). Specifically, cells were co-infected with AAV2 (MOI 100) and either HSV-1 (MOI 0.1) or AdV5 (MOI 0.1). After 48 h, the cells were harvested, and extrachromosomal DNA was extracted using the Hirt protocol (39). To minimize the background of input AAV2 DNA, the MOI was kept low. Sequencing results showed different DNA replication intermediates that were classified according to the previously described categories (i) monomers, (ii) dimers, (iii) head-to-tail (HT) repeats, (iv) alternating repeats, (v) HT and alternating repeats, (vi) ITR repeats, and (vii) others that could not be classified in one of the categories 1–6 (38). HT repeats are indicative of RCR, while alternating repeats may arise from RHR in the absence of terminal resolution. The results are shown in Table 1 and can be summarized as follows: the majority of reads consisted of AAV2 genome monomers in both HSV-1 (33%) and AdV5 (29%) supported replication. There was an approximately fivefold higher percentage of HT repeats (11%) compared to alternating repeats (2%) in HSV-1 co-infected cells. Cells co-infected with AdV5 showed more alternating repeats (2%) than HT repeats (1%). Interestingly, AdV5 co-infection induced the generation of ITR repeats much more efficiently (29.6%) than HSV-1 co-infection (2.4%).

**TABLE 1:** Read analysis of sequencing data from cells co-infected with AAV2 (MOI 100) and either HSV-1 (MOI 0.1) or AdV5 (MOI 0.1) at 48 hpi^*a*^

Category	1	2	3	4	5	6	7
	Monomer	Duplex	Head-to-tail repeats	Alternating repeats	Head-to-tail and alternating repeats	ITR repeats	Others
Sample	Ratio	Ratio	Ratio	Ratio	Ratio	Ratio	Ratio
AAV2/HSV-1	0.330	0.256	0.110	0.024	0.020	0.024	0.236
AAV2/AdV5	0.290	0.130	0.010	0.020	0.004	0.296	0.250

^*a*^ Table shows ratios of the different categories of AAV2 DNA replication intermediates.

### Southern analysis confirms differential AAV2 DNA replication mechanisms depending on the helper virus

**Fig 2:**
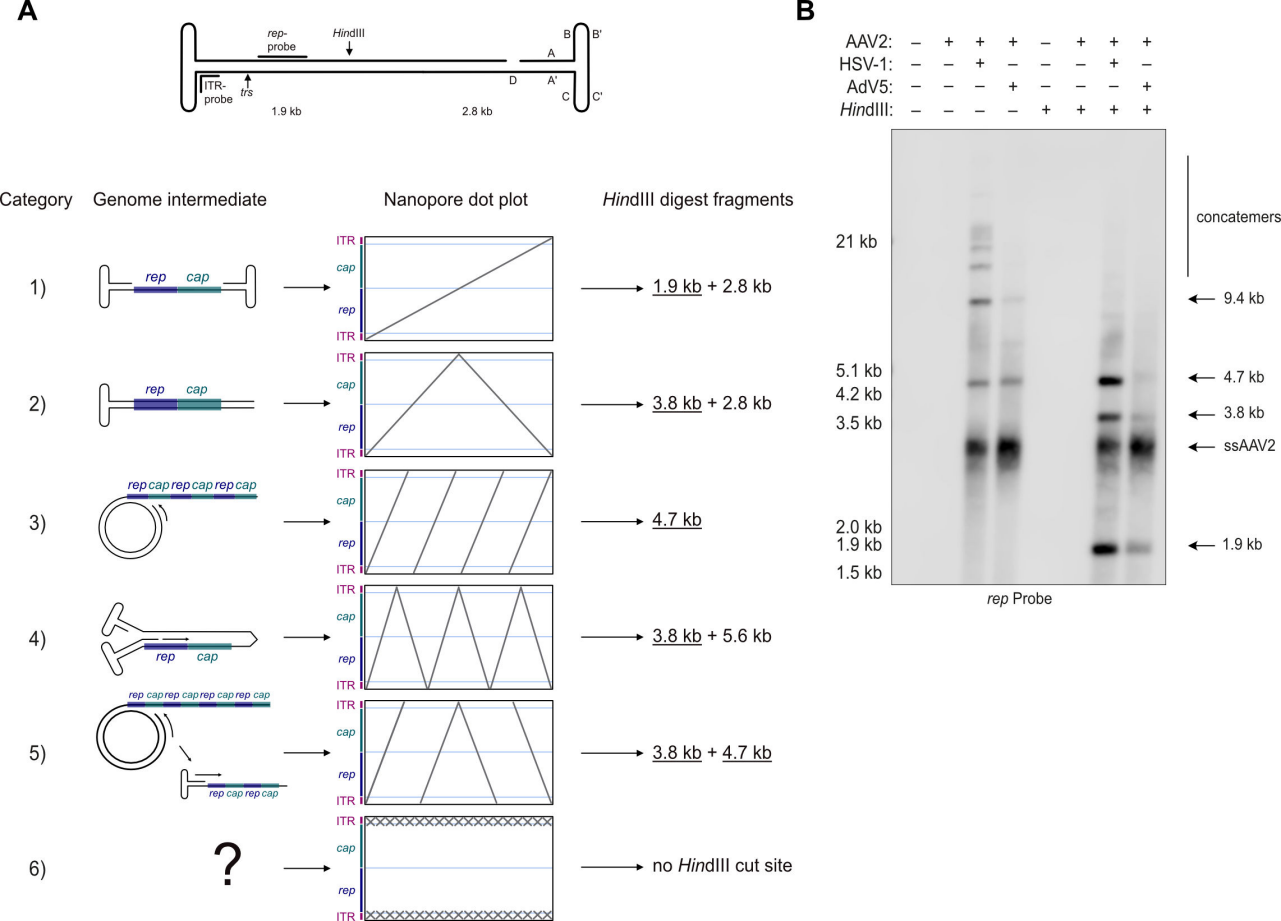
Southern blot of AAV2 DNA replication intermediates.(**A**) Schematic representation of the AAV2 genome with ITR regions, *trs*, *Hin*dIII restriction site, and probe-binding sites indicated (top). Schematic representation of the different categories of expected Nanopore dot plots and corresponding genome intermediate. Expected fragment sizes after *Hin*dIII digest are indicated. Underlined sizes indicate fragments that can be observed on the Southern blot due to the location of the Rep probe. (**B**) Southern blot of untreated or *Hin*dIII treated Hirt DNA extracted from Vero cells infected with AAV2 (MOI 100) or co-infected with AAV2 (MOI 100) and either HSV-1 (MOI 0.1) or AdV5 (MOI 0.1) at 48 hpi. Bands were visualized with a *rep*-specific probe.

AAV2 replication intermediates were also investigated by a Southern blot of Hirt DNA extracted from cells co-infected with AAV2 and either HSV-1 or AdV5. Some of the samples were treated with *Hin*dIII to investigate the nature of the concatemeric replication intermediates. *Hin*dIII cuts the ds AAV2 genome within the *rep*-sequence, specifically at nucleotide position 1882 (NC_001401, Fig. 2A) and yields the following predicted DNA fragments: 1.9 and 2.8 kb for monomers, 3.8 and 5.6 kb for head-to-head (HH) concatemers, and 4.7 kb for HT concatemers. Because of the specificity of the hybridization probe (Fig. 2A), only the bands at 1.9, 3.8, and 4.7 kb can be detected. In both untreated samples, bands at approximately 3 kb, which represent the ss AAV2 genome, and at approximately 4.7 kb and 10 kb, which correspond to ds monomeric and dimeric AAV2 DNA, respectively, were observed (Fig. 2B). Higher molecular weight bands representing AAV2 genome concatemers were observed only in the presence of HSV-1 as the helper virus. *Hin*dIII treatment resulted in the disappearance of all bands representing ds dimers and higher-order multimers of the AAV2 genome. Instead, a strong band at 4.7 kb, which is expected from cleavage of HT-linked concatemers that arise from RCR, appeared in the Hirt DNA prepared from AAV2 and HSV-1 co-infected cells. Additional bands appeared at 3.8 and 1.9 kb which are consistent with cleavage of HH-linked concatemers arising from RHR and of ds AAV2 genome monomers, respectively. In the samples from AAV2 and AdV5 co-infected cells, *Hin*dIII cleavage produced strong bands at 3.8 kb, which is expected from cleavage of HH-linked concatemers, and at 1.9 kb, which is expected from cleavage of ds AAV2 monomers. A much weaker band at 4.7 kb may also represent a cleavage product from HT-linked concatemers.

Southern blotting and Oxford Nanopore sequencing experiments in HeLa and BJ cells revealed that HSV-1-supported AAV2 genome replication results in higher ratios of concatemeric AAV2 DNA compared to AdV5. At 48 hpi, higher-molecular-weight bands, and specific band patterns after *Hin*dIII digestion indicated distinct AAV2 replication mechanisms between the helper viruses, corroborating preferential RCR in HSV-1 co-infections and RHR in AdV5 co-infections across different cell types and MOIs (Fig. S1A to D). Nevertheless, differences in ratio of ITR repeats in AAV2 and AdV5 co-infection indicate that AdV5 co-infection differs more substantially across different cell types compared to HSV-1 co-infection (Fig. S1D).

Overall, the data corroborate the sequencing data and indicate that AAV2 replicates predominantly using the RCR mechanism in the presence of HSV-1 and the RHR mechanism in presence of AdV5.

### AdV5 co-infection leads to the generation of vast amounts of ITR repeats

**Fig 3:**
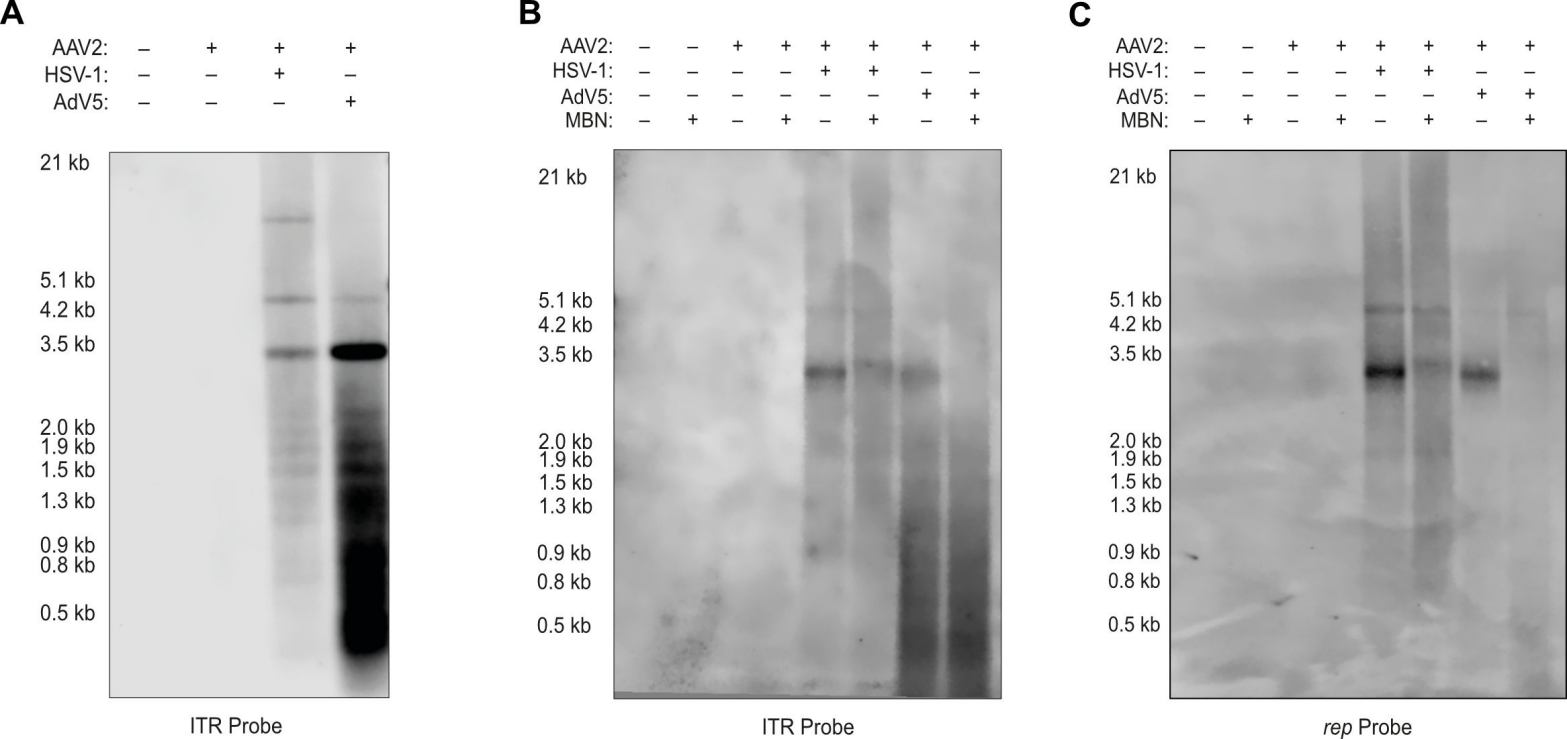
Southern analysis of ITR repeats in total cells or supernatant. Southern blot of Hirt DNA extracted from (**A**) total cells or (**B and C**) supernatant of cells infected with only AAV2 (MOI 100) or co-infected with AAV2 (MOI 100) and HSV-1 (MOI 0.1) or AdV5 (MOI 0.1) at 48 hpi. Bands were visualized with either (**A and B**) an ITR-specific probe or (**C**) a *rep-*specific probe.

To exclude the possibility that the ITR repeats identified by Oxford Nanopore sequencing (Table 1) are due to a sequencing artifact, we designed a hybridization probe specific for the C and A sequence within the ITR (Fig. 2A). Southern analysis showed robust staining of low-molecular-weight bands in samples from AAV2 and AdV5 co-infected cells. These bands were observed also in samples from AAV2 and HSV-1 co-infection but with a much lower intensity. Distinct further bands at approximately 3 and 4.7 kb were detected in both co-infections and may represent ss and ds AAV2 DNA monomers, respectively. An additional band of approximately 10 kb was observed in the sample from AAV2 and HSV-1 co-infected cells only and may represent the ds dimer of the AAV2 DNA (Fig. 3A).

Interestingly, using the ITR-probe (Fig. 3B) but not the *rep* probe (Fig. 3C), strong staining of sub genome-length low-molecular-weight, Mung bean nuclease (MBN)-resistant DNA was observed when DNA was extracted from progeny AAV2 particles purified from AAV2 and AdV5 co-infected cells. This shows that the ITR repeats are not an artifact from Oxford Nanopore sequencing library preparation but more likely a byproduct of the AAV2 genome replication process.

### ITRs in head-to-tail concatemers show the same orientation

**Fig 4:**
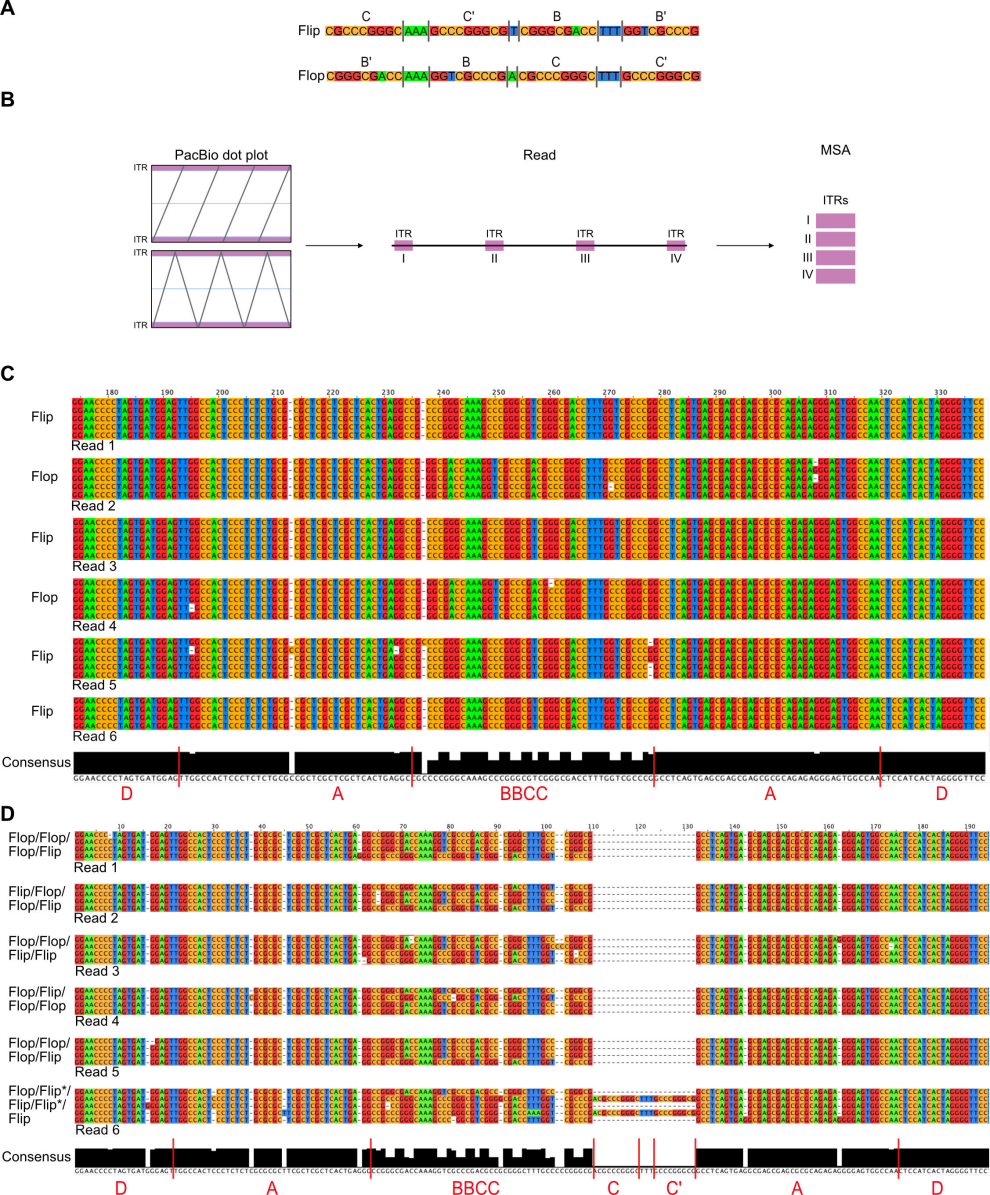
MSA analysis of ITR regions.(**A**) Schematic representation of the “flip” and “flop” orientation of the BBCC region of the ITR. Colors represent different bases: C, orange; G, red; A, green; T, blue. (**B**) Schematic representation of RCR with cAAV as substrate. Expected ITR orientation in RCR product is indicated based on ITR orientation of the cAAV substrate. The ITR regions of the six longest (**C**) HT repeats and (**D**) alternating repeats from HSV-1 co-infection were aligned and the orientations were indicated. Each block represents one read.

To further investigate, whether HT concatemers of the AAV2 genome are a product of RCR, the nucleotide sequence was examined in greater detail. Hirt DNA from cells infected as described above was analyzed using PacBio sequencing, as this method offers higher accuracy on a single base level compared to Oxford Nanopore sequencing (40, 41). The sequencing data revealed that the ITR regions joining two AAV2 genomes in the concatemeric DNA (HT and alternating repeats) consisted of a modified ITR (TRT) containing an additional flanking D sequence (Fig. 4C and D), which has been previously described in circular AAV genomes isolated from infected cells (3). As previously mentioned AAV ITRs can occur in two different orientations, “flip” and “flop” (Fig. 4A), with their relative positioning independent of each other (2, 33). Assuming HT concatemers are the product of RCR with circular AAV2 (cAAV2) as the substrate, it would be expected that ITRs within one read would show the same orientation. Multiple sequence alignment (MSA) of six HT reads (Fig. S2A) with the ITRs contained in one read aligned (Fig. 4B), showed that the ITRs occur, indeed, exclusively in either a “flip” or a “flop” orientation in one read. This further supports RCR as a mechanism for HT concatemers with cAAV2 as the substrate. The ITRs in alternating repeats (Fig. S2B) showed a random distribution of orientation (Fig. 4D). This finding is consistent with RHR in the absence of *trs* resolution, as within one AAV genome the orientation of the ITRs is random and independent of each other (33). Interestingly, one read with alternating repeats was found, showing an additional CC′ sequence in two of the five ITR regions within the read (Fig. 4D).

### The contribution of recombination to the formation of AAV2 genome concatemers is negligible

**Fig 5:**
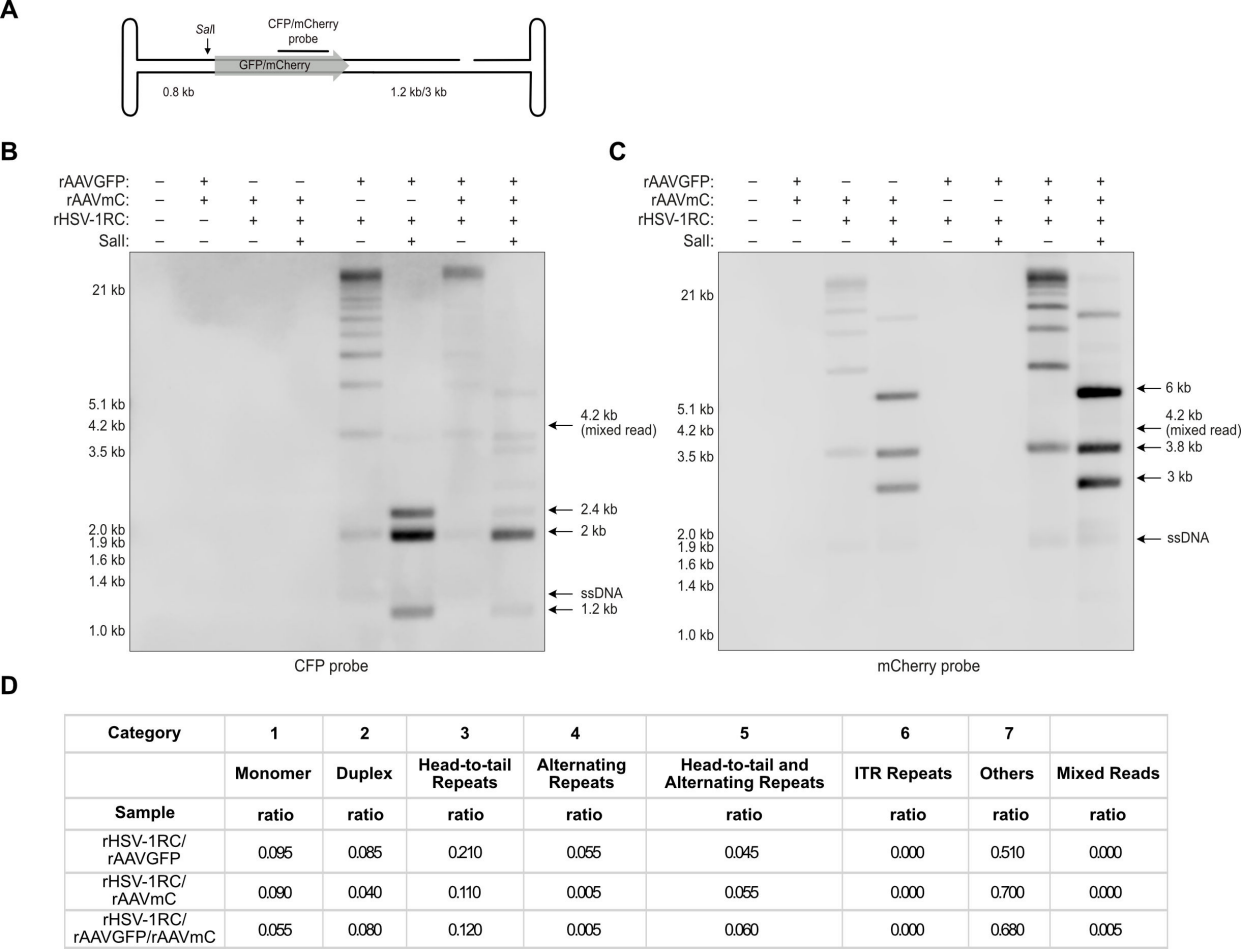
Southern blot and Oxford Nanopore read analysis of recombination efficiency. (**A**) Schematic representation of the rAAV genome with *Sal*I cut site, expected fragment sizes, and probe-binding site indicated. Transgenes are indicated as gray arrow. Southern blot of untreated or *Sal*I-treated Hirt DNA from cells infected with rAAVGFP (MOI 100, 2 kb), rAAVmC (MOI 100, 3.8 kb), and rHSV-1RC (MOI 0.1) in indicated combinations at 48 hpi. Sizes of HH and HT concatemers or monomer fragments in *Sal*I-treated samples are indicated with arrows. The arrow at 4.2 kb indicates the predicted size of a *Sal*I digested mixed fragment. AAV2 DNA was visualized with either (**B**) a CFP probe or (**C**) a mCherry probe. (**D**) Read analysis of sequencing data of cells infected as described above. The table shows the ratio of the different categories of a total of 200 reads.

**Fig 6:**
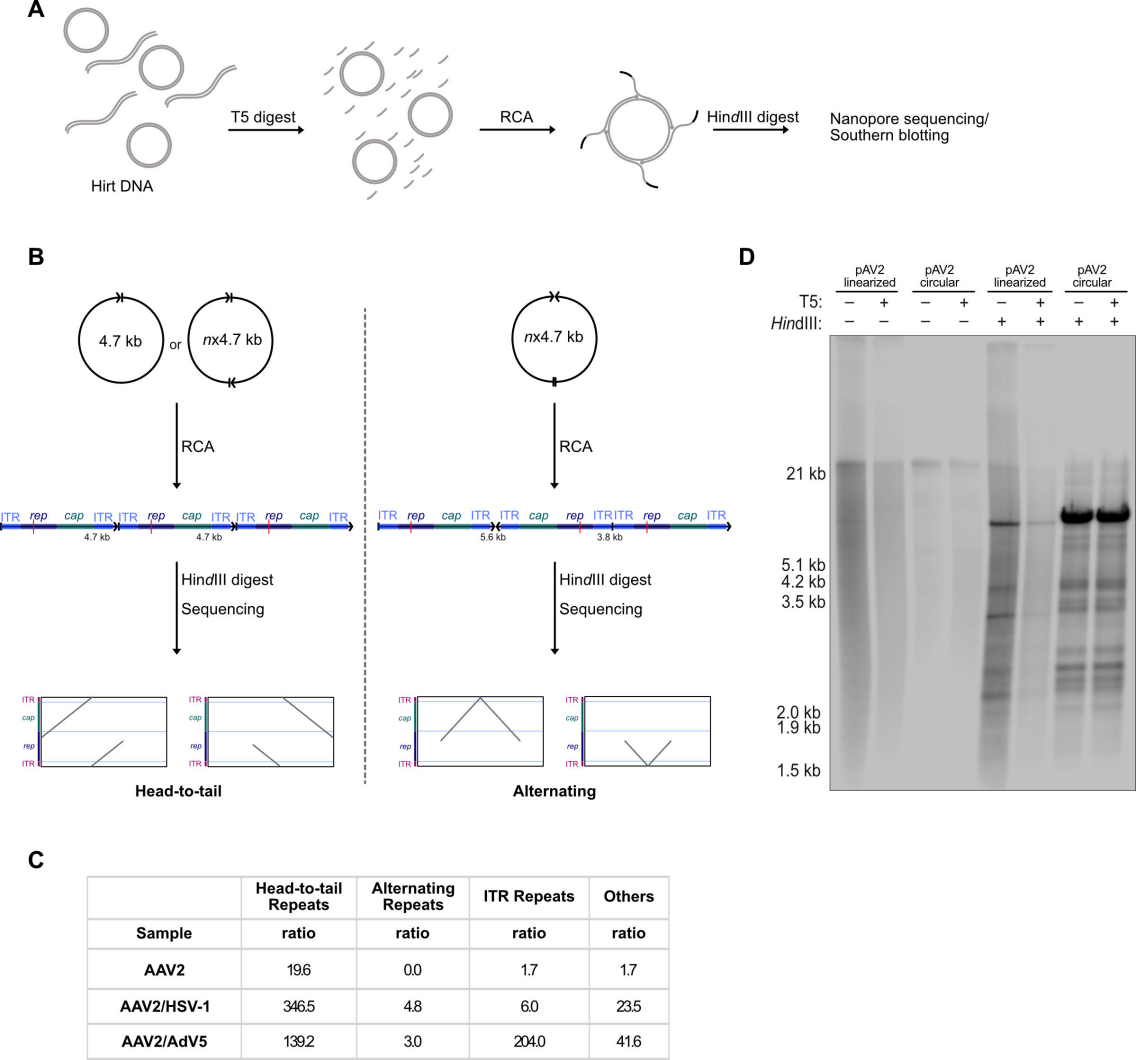
Analysis of circular AAV2 genomes in HSV-1 or AdV5 co-infected cells. (**A**) Schematic detailing of the experimental steps. Hirt DNA from cells infected with AAV2 (MOI 100) alone or co-infected with AAV2 and either HSV-1 (MOI 0.1) or AdV5 (MOI 0.1) was treated with T5 exonuclease to digest linear genomes. Circular genomes were amplified using RCA and subsequently treated with *Hin*dIII prior to sequencing or Southern blotting. (**B**) Schematic showing the expected plots from Oxford Nanopore sequencing after *Hin*dIII digest. Left indicates products expected from a circular monomer or HT circular AAV2 genome, and right indicates products expected from an alternating circular AAV2 genome. (**C**) Read analysis of sequencing data of cells infected and treated as described above. For normalization, ratios describe reads per 10,000 mitochondrial reads. (**D**) Southern blot of control digest of pAV2 plasmid as described above, showing T5 digest and RCA product after *Hin*dIII digest. Bands were visualized using a *rep*-specific probe.

AAV2 genome concatemers could arguably also arise from genome recombination events. However, the probability of recombination leading to the formation of long, HT concatemers of the AAV2 DNA would be exceedingly low, as previously discussed by Meier et al. (38). Nevertheless, to show if and to what extent recombination plays a role in HSV-1 supported AAV2 genome replication, Hirt DNA of cells infected with two different recombinant AAV vectors (rAAV), encoding for either GFP (rAAVGFP) or mCherry (rAAVmC) and co-infected with a recombinant HSV-1 encoding Rep and Cap (rHSV-1RC, Fig. S3) to support rAAV replication were analyzed by Southern blot (Fig. 5A through C,). Four regions with lengths ranging from 120 to 700 bp are virtually identical between the two different rAAV vector genomes and would readily support homologous recombination. To reveal the nature of the concatemers, some samples were treated with *Sal*I which cuts the ds rAAV genomes at nucleotide position 815 (Fig. 5A) and yields the following predicted DNA fragments: 0.8 and 1.2 kb from monomers of rAAVGFP or 0.8 and 3 kb from monomers of rAAVmC, of which only the bands at 1.2 and 3 kb can be detected because of the specificity of the hybridization probe. *Sal*I digestion of HT concatemers of rAAVGFP and rAAVmC genomes would yield detectable bands at 2 kb and 3.8 kb, respectively. *Sal*I digestion of HH concatemers of rAAVGFP and rAAVmC genomes would yield detectable bands at 2.4 kb and 6 kb, respectively. Cleavage of a recombined, mixed HH concatemer, would yield a band at 4.2 kb. Cleavage of a recombined, mixed HT concatemer would yield bands at 2 kb or 3.8 kb, but these could not be differentiated from *Sal*I digested HT concatemers of rAAVmC or rAAVGFP. Fig. 5B and C shows that co-infections with each rAAV individually or in combination and the helper virus yielded the expected patterns for the respective vector genomes, including high-molecular-weight concatemers. S*al*I treatment resulted in the disappearance of all bands representing ds dimers and higher-order multimers of the rAAV genomes. Instead, strong bands at 2 (Fig. 5B) and 3.8 kb (Fig. 5C), which are expected from cleavage of RCR products, appeared. Additional bands appeared at 2.4 (Fig. 5B) and 6 kb (Fig. 5C), which are consistent with RHR products. Importantly, at 4.2 kb, the expected size for a recombined HH fragment, no band was detected with either probe.

As recombination levels might be below the detection limit of the Southern blot, we additionally investigated recombination on a single genome level using Oxford Nanopore sequencing. Cells were infected as described above, and Hirt DNA was prepared and sequenced. Similar to the co-infections with wild-type (wt) AAV2, in all co-infections with rAAVs, more HT repeats (21%, 11%, and 12%) were present compared to alternating repeats (5.5%, 0.5%, and 0.5%) (Fig. 5D; Fig. S3), indicating that replication preferentially occurs by RCR. The mixed reads in cells co-infected with rAAVGFP, rAAVmC, and HSV-1RC showed a ratio 0.5% indicating that recombination plays a minimal role in concatemer formation. Interestingly, when using rAAV instead of wt AAV2 in co-infections with a helper virus, most reads belonged to category 7 and no ITR repeats were observed (Fig. 5D). Additionally, recombination rates in AdV5 supported AAV2 genome replication amounts to 0.03% (Fig. S4).

### HSV-1 co-infection leads to a higher AAV2 circularization ratio compared to AdV5 co-infection

Besides recombination alternating repeats could also be explained by RCR, provided the cAAV2 substrate consists of two AAV2 genomes in alternating orientation. To investigate the nature of cAAV2 in AdV5 and HSV-1 infected cells, Hirt DNA from cells infected as described previously was treated with T5 exonuclease to remove linear AAV2 DNA and amplified by rolling circle amplification (RCA) to increase circular DNA yield. After RCA, the DNA was treated with *Hin*dIII to reveal the orientation (Fig. 6A). Circular monomers or HT joined cAAV2 would lead to parallel lines on the plot and alternatingly joined cAAV2 would lead to a “V” or inverted “V” shape, both starting and ending at the *Hin*dIII site (Fig. 6B). Sequencing data revealed a higher ratio of HT repeats in HSV-1 supported AAV2 genome replication (346.5) compared to AdV5 co-infection (139.2), whereas alternating repeats showed comparable ratios in HSV-1 (4.8) or AdV5 (3.0) co-infections (Fig. 6C), indicating that cAAV2 is mostly present either in monomer or HT-linked form and further supporting preferential RCR when HSV-1 is the helper virus and RHR when AdV5 is the helper virus. Interestingly, ITR repeats were also found in the sequencing results, suggesting that ITR repeats may arise from a circular substrate or are resistant to T5 digest. Consistent with previous observations (Table 1), AdV5 co-infection leads to a higher ratio of ITR repeats (204.0) compared to HSV-1 co-infection (6.0) (Fig. 6C).

As proof of concept a circular plasmid containing the AAV2 genome (pAV2) was either linearized or kept circular prior to T5 exonuclease or mock treatment. Following T5 treatment, RCA was performed, digested with *Hin*dIII, and analyzed by Southern blotting. Both circular samples (±T5) showed strong amplification, while the linearized, mock, and T5-treated samples showed weak bands (Fig. 6D). This confirms that DNA sequencing after T5 treatment and RCA is appropriate to determine the nature of cAAV2.

### The HSV-1 polymerase is essential in AAV2 genome concatemer formation

**Fig 7:**
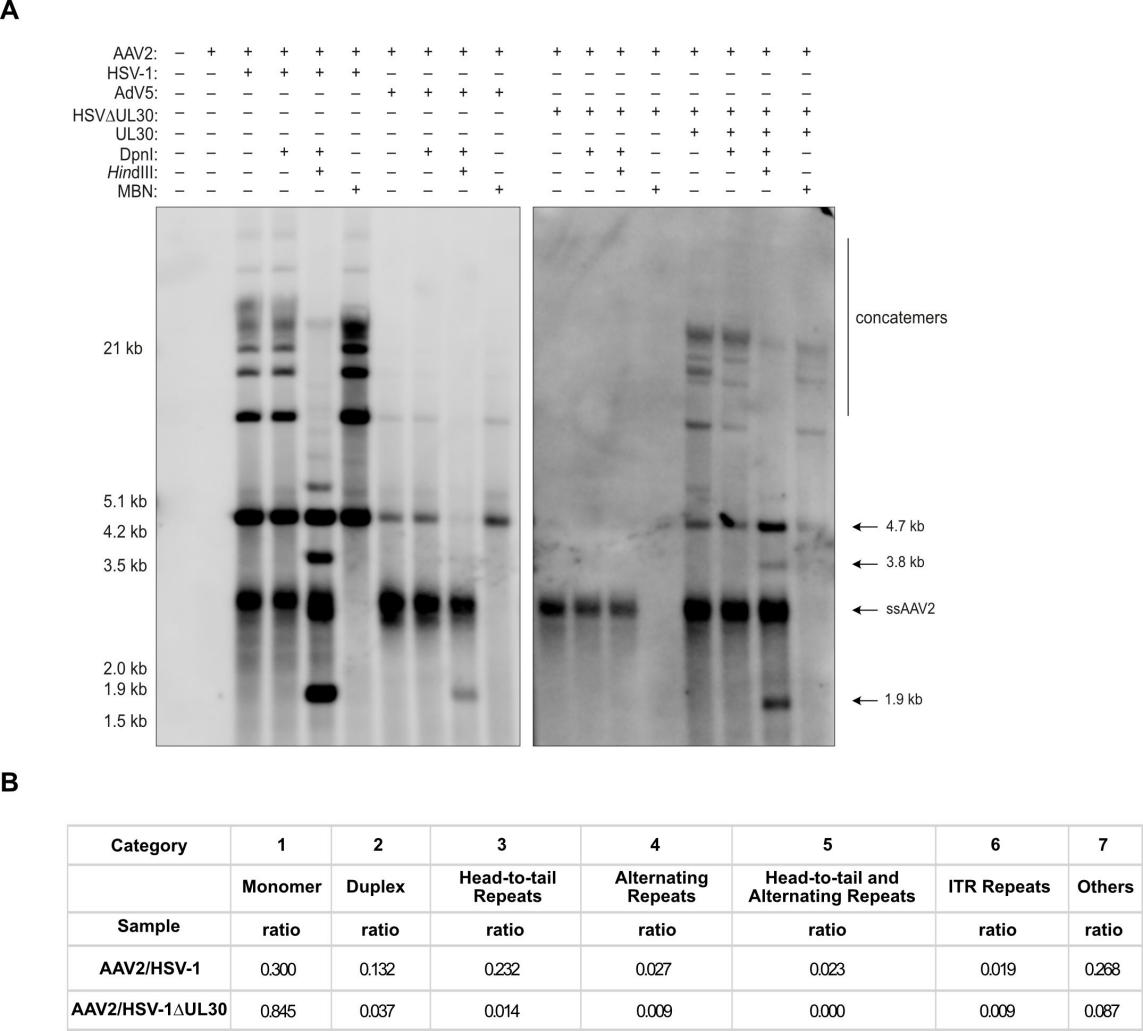
Southern blot and Oxford Nanopore read analysis of AAV2 DNA replication intermediates. (**A**) Southern blot of untreated or *Hin*dIII treated Hirt DNA from cells infected with AAV2 or co-infected with AAV2 (MOI 100) and HSV-1 (MOI 0.1), HSV-1∆UL30 (MOI 1), or HSV-1∆UL30 (MOI 1) with reconstituted UL30. (**B**) Sequencing read analysis of Hirt DNA from cells co-infected with AAV2 and HSV-1 or HSV-1∆UL30. The table shows the ratio of the different categories of a total of 200 reads.

Depending on the helper virus supporting AAV replication, different cellular and viral proteins are recruited to AAV2 replication compartments (42, 43). The HSV-1 polymerase is not strictly necessary for AAV replication but was demonstrated to strongly enhance it (18, 19, 44). As HSV-1 replicates its DNA using a RCR mechanism and requires HT concatemers for packaging (13), we investigated the possibility that the HSV-1 polymerase is involved in the formation of HT repeats of the AAV2 genome. For this, cells were co-infected with either wt HSV-1 or an HSV-1 mutant (HSV∆UL30) in which the catalytic subunit (UL30) of the HSV-1 polymerase complex is non-functional. In a second step, cells infected with HSV∆UL30 were reconstituted by transfection of an UL30 encoding plasmid. The results of Southern analysis and Oxford Nanopore sequencing, indeed, imply an important role for the HSV-1 polymerase in concatemer formation, as HT concatemers of the AAV2 genome were not found in the presence of HSV∆UL30 as the helper virus (Fig. 7A and B) but were detected after reconstitution of UL30 (Fig. 7A).

## DISCUSSION

In the presence of AdV as the helper virus, AAV DNA replication is assumed to proceed in an RHR mechanism (30, 35, 45, 46). However, AAV may adapt its replication process depending on the cellular environment and the helper virus, as we recently showed that HSV-1 co-infection facilitates RCR of the AAV2 genome (38). Here, we directly compared the formation of AAV2 DNA replication intermediates in the presence of the two different helper viruses and analyzed the junctions between concatemeric genomes in detail. The sequencing data as well as the results from Southern blotting revealed a much higher ratio of HT concatemers, the products of RCR, compared to alternating repeats, the products of RHR, in HSV-1 co-infection while the opposite was true in AdV5 co-infection.

The formation of divergent sets of AAV2 DNA replication products is likely due to the different helper functions provided. The essential helper factors from AdV include E1a (22), E1b55k (25), E2a (47), E4orf6 (48), and the VA RNA (27); the essential HSV-1 helper factors consist of the helicase-primase (HP) complex, composed of UL5/UL8/UL52, and the ssDNA binding protein ICP8 (17). Interestingly, neither the AdV DNA polymerase nor the HSV-1 DNA polymerase is required for AAV genome amplification, but the HSV-1 DNA polymerase was shown to strongly enhance AAV genome replication (18, 19, 44). The HSV-1 DNA polymerase together with ICP8 and the HP complex can confer RCR to circular plasmids even in the absence of an HSV-1 origin of DNA replication or the HSV-1 origin-binding protein (49). The AAV DNA has, indeed, been shown to readily circularize within the host cell (50–52) thereby provides a potential template for RCR. Moreover, the cAAV genomes contain a TRT which constitutes a functional origin for AAV DNA replication (3). Our sequencing data revealed that the AAV2 genomes in the HT concatemer are not only joined by TRTs, but also that all TRTs in one molecule have the same orientation, either all “flip” or all “flop.” These two observations concerning the TRTs further support that AAV2 DNA synthesis proceeds as an RCR mechanism, at least in the presence of HSV-1, and suggest that cAAV is the most likely substrate. Interestingly, cAAV2 was found more abundantly in the presence of HSV-1 compared to AdV5 which provides further evidence that the AAV2 genome replicates preferentially with a RCR mechanism when HSV-1 is the helper virus and preferentially with an RHR mechanism when AdV5 is the helper virus.

While AdV5 and HSV-1 have different growth kinetics (Fig. S5) and display variations in AAV2 gene expression levels (Fig. S6), AAV2 viral titers show non-significant differences (Fig. S5A). Nevertheless, both helper viruses provide sufficient helper functions for AAV2 genome replication to be able to discern the differences in replication mechanisms (Fig. 1 and 2B) and show comparable results with 10 times higher MOI of the helper viruses (Fig. S1A).

High-molecular-weight AAV2 genome concatemers were only detected in HSV-1-supported AAV2 genome replication but not in AdV5-supported replication. This might be possibly due to AdV5 proteins that are able to counteract concatemer formation. For example, AdV E4orf6 has been shown to stabilize the AdV genome and prevent unwanted concatemerization. During AdV infection, E4orf6 in complex with E1B55k inhibits p53-mediated anti-viral responses and non-homologous end joining (NHEJ)-dependent processes of concatemerization of the AdV genome through targeted degradation of p53 and meiotic recombination 11 (MRE11) (53, 54). As an essential helper factor in AAV genome replication (48, 55), AdV E4orf6 may have a similar role in the inhibition of AAV2 genome concatemerization. However, our finding that with either helper virus the contribution of recombination to the formation of AAV genome concatemers is negligible argues against this possibility as E4orf6 blocks concatemer formation via blocking NHEJ-mediated recombination (53, 54).

Alternatively, the lack of HT concatemers of the AAV2 genome in the presence of AdV5 might not be due to a different replication mechanism but rather due to more efficient terminal resolution, which prevents concatemer formation.

The question remains whether HT concatemers can yield single-stranded, packageable, and infectious AAV genomes and/or contribute to AAV infection as templates for viral gene transcription. Packaging into AAV capsids would require the excision from a concatemer of a single-stranded monomeric AAV2 genome containing a complete ITR at each end. A mechanism that yields such genomes from concatemers is, indeed, conceivable if considering that cAAV containing a single TRT represents the simplest form of a HT concatemer. Musatov et al. (56) demonstrated that cAAV can give rise to packageable AAV genomes and proposed the following model for this: Rep68/78 can bind to the A sequence and initiate RCR by nicking at the *trs*. This is followed by an extension of the newly generated 3′ end and simultaneous strand displacement. After the replication fork has completed a full circle, the displaced strand is cleaved by Rep, resulting in a single-stranded monomer with a complete ITR on the 5′-end and a 3′-end with only a D sequence. The incomplete ITR can be repaired via a single-stranded panhandle intermediate formed by annealing of two inverted D sequences and extension of the incomplete strand, a mechanism that has, indeed, been described for AAV (5).

There is a striking difference in the abundance of AAV2 ITR repeats formed in the presence of HSV-1 and AdV5. Interestingly, the ITR repeats were also found in AAV2 progeny, encapsidated or bound to the outside of the capsid, and may, therefore, play a role early in AAV2 infection. For example, AAV ITRs can activate the DNA damage response (DDR) involving Ataxia-Telangiectasia Mutated (ATM) and RAD3-related (ATR) kinases that inhibit cell cycle progression and lead to apoptosis in a p53-dependent manner (57, 58). Extra-viral DNA has been shown to induce Toll-like receptor 9 (TLR9)-dependent immune responses in human plasmacytoid dendritic cells, which was inhibited by DNase treatment of the AAV stocks (59).

Proteins of the DDR may be involved in the formation of ITR repeats as DNA-dependent protein kinase catalytic subunit (DNA-PKcs) and Artemis-associated endonuclease (Artemis) have been shown to cleave ITR hairpins of rAAV vectors in a tissue-dependent manner (60). Besides DNA-PKcs, Artemis can also be activated through ATM (61). The resulting structure can be cleaved at the *trs* by Rep68/78 and the newly generated 3′-end can be extended, resulting in a fragment containing two ITRs. Subsequently, the ITRs can reanneal, forming a double hairpin structure and thereby providing self-priming activity. The 3′-end can then again be extended. Following this mechanism, it is conceivable that the ITRs cleaved by DNA-PKcs and Artemis can serve as substrate for the observed ITR repeats. The difference in ITR repeat ratios in AdV5 vs HSV-1 supported AAV2 genome replication may be due to differences in DNA-PKcs levels, as this kinase was found to be the primary mediator of damage signaling in response to AAV genome replication in AdV co-infection (62). In HSV-1 co-infection, DNA-PKcs was shown to be degraded in an ICP0-dependent manner (63, 64) although at a delayed rate in co-infection with AAV2 (65). This delay may be important for HSV-1-supported RCR of the AAV genome, as AAV genome circularization, a pre-requisite for RCR (66), was shown to be impaired in DNA-PKcs-deficient SCID mice (67–69). Furthermore, the ability to induce a significant DDR was linked to the p5 region on rAAV2 vectors as vectors lacking the p5 region of AAV2 were not capable of inducing such a response (70). Indeed, in infections with rAAV2s, that do not contain the p5 region, we did not find ITR repeats, further linking the formation of ITR repeats to the DDR.

The ITR repeats, but also category 7 (others), is reminiscent of defective viral genomes. Defective viral genomes have been shown to play a role during the viral life cycle, for example, to induce high level of interferon (IFN) during infection and/or promote viral persistence (reviewed in reference (71)). One class of defective viral genomes are copy-back or snap-back genomes, which were abundantly present in category 7 of the sequencing data. Interestingly, a study by Zhang et al. demonstrated that copy-back AAV genomes containing parts of either *rep* or *cap* contributed to the regulation of AAV gene expression and improved packaging. However, excess presence of these copy-back genomes was shown to have an inhibitory effect (72). It is conceivable that the ITR repeats play a yet unidentified role in the viral life cycle as well.

In this study, we provide evidence that AAV2 can adapt its replication mechanism depending on the helper virus. Several differences in the ratios of specific replication intermediates such as concatemers and ITR repeats were found. The biological relevance of these needs further investigations. Besides different helper factors, differences in Rep levels/functions may impact the replication mechanism. The Rep68/78 endonuclease activity or its phosphorylation may be differentially regulated depending on the helper virus. Further understanding of the replication mechanism may contribute to improve current systems for rAAV production, potentially increase rAAV yields and possibly inhibit undesired secondary responses in the host during treatment with rAAV vectors.

## MATERIALS AND METHODS

### Cells and viruses

Vero cells (African green monkey kidney) were obtained from ATCC (Manassas, Virginia, USA) and cultured in Dulbecco’s modified Eagle medium (DMEM) supplemented with 10% fetal bovine serum (FBS), 100 µg/mL streptomycin, and 100 units/mL penicillin in a humified incubator at 37°C and 5% CO_2_. Vero 2–2 cells (73) were cultured in DMEM supplemented with 10% FBS, 100 µg/mL streptomycin, 100 units/mL penicillin, and 500 µg/mL of G418 (G418 Sulfate, 10131035, Thermo Fisher Scientific, Waltham, MA, USA) in a humified incubator at 37°C and 5% CO_2_. Stocks of wt HSV-1 (strain F, provided by B. Roizman, University of Chicago) and HSV∆UL30 (HP66, provided by D. Coen, Harvard University, Boston, MA, USA) (74) were produced as previously described (75). Purified wt AAV2 was produced by the Viral Vector Facility (University of Zurich, Switzerland) as previously described (65, 76) using the pAV2 plasmid (77). The recombinant AAV2 genome rAAVeCFPrep was constructed by replacing the mCherry coding sequence in the plasmid pAAVtCR (18) with the ECFP coding sequence from pECFP (Clontech, Mountain View, CA, USA). Purified recombinant AAV2 vectors, rAAVGFP, rAAVeCFPrep, and rAAVmCherry (kindly provided by S. Sutter, University of Zurich, Switzerland), were produced by transient transfection of 293T cells with pDG (78) and pAAVeGFP (kindly provided by M. Linden, King’s College London School of Medicine, London, UK), pAAVeCFPrep or pAAVmCherry (kindly provided by J. Neidhardt, University of Zurich, Switzerland) and purified by an iodixanol density gradient. AdV5 was provided by U. Greber/M. Suomalainen (University of Zurich, Department of Molecular Life Sciences, Switzerland).

### Infection protocol

Cells were seeded the day before at 2 × 10^6^ cells per 10 cm tissue culture plate. For infection, the cell culture medium was replaced with virus inoculum [viruses and corresponding multiplicity of infection (MOI) are indicated in the figure legends]. After allowing the virus to adsorb for 30 min at 4°C, the cells were placed in a humified incubator for 1 h at 37°C and 5% CO_2_. Then, the virus inoculum was replaced with DMEM containing 2% FBS, and the cells were further incubated for the timepoints indicated in each experiment.

For combined infection and transfection experiments, the cells were infected as described above and, after the 1 h adsorption period at 37°C, transfected using Lipofectamine 2000 (11668019, Thermo Fisher Scientific, Waltham, MA, USA) with 10 µg of pCM-pol encoding HSV-1 UL30 or 10 µg of pCM-pol and 10 µg of pCM-UL42 encoding HSV-1 UL42 (Heilbronn, 1989, JVI) per plate according to the protocol provided by the supplier. At 24 h post infection (pi), FBS was added to reach a concentration of 2% in the medium, and the cells were further incubated at 37°C and 5% CO_2_ for 24 hpi.

### Southern blot

Extrachromosomal DNA was extracted from infected cells using the Hirt protocol (39) and either digested extensively with *Hin*dIII, *Sal*I, or mung bean nuclease (MBN) (R0104S, R0138S or M0250S, New England Biolabs, MA, USA) or left untreated. The DNA was separated on 0.8% agarose gels and transferred onto nylon membranes (Hybond-N+, RPN203B, Amersham, Little Chalfont, UK). As size reference, DIG-labeled marker DNA was used (DNA molecular weight marker III, 11218602910, Roche). Hybridization was performed with probes specific for AAV2 *rep*, AAV2 ITR, ECFP/EGFP, or mCherry sequences. Detection with anti-digoxigenin antibody conjugated with alkaline phosphatase (Anti-Digoxigenin-AP Fab fragments, 11093274910, Roche, Switzerland) and activation with the chemiluminescent substrate CDP-Star (11759051001, Roche, Switzerland) was performed according to the manufacturers’ protocols. The *rep*, CFP/GFP, and mCherry probes were synthesized using the PCR digoxigenin probe synthesis kit (1163090910, Roche, Switzerland) with primers shown in Table 2 and the following PCR conditions: 95°C for 2 min, followed by 95°C for 30 s, 55°C (ECFP/EGFP and mCherry) or 60°C (*rep*) for 30 s, and 72°C for 40 s for 30 cycles. The AAV2 ITR-specific probe was labeled using the DIG DNA labeling kit (11175033910, Roche, Switzerland) using the following oligonucleotide: 5′-ttt ggt cgc ccg gcc tca gtg agc gag cga gcg cgc aga gag gga gtg gcc aa-3′. Chemiluminescence was visualized with the LI-COR imaging system Odyssey Fc (LI-COR Biosciences, NE, USA).

**TABLE 2:** Primer sequences used for Southern blot probe synthesis^*a*^

Probe	Forward primer	Reverse primer	Probe length	Hybridization temperature
*rep*	5′-gaa cgc gat atc gca gcc gcc atg ccg gg-3′	5′-gga tcc gaa ttc act gct tct ccg agg taa tc-3′	735 bp	48.9°C
CFP/GFP	5′-ctg acc ctg aag ttc atc tgc-3′	5′-gga tct tga agt tgg cct tg-3′	378 bp	50.3°C
mCherry	5′-agg acg gcg agt tca tct ac-3′	5′-ttc cac gat ggt gta gtc ctc-3′	295 bp	51.1°C

^*a*^ Table indicates the generated probe length and hybridization temperature for each probe.

### T5 exonuclease assay

Hirt DNA was extracted from infected cells using the Hirt protocol (39), and linear DNA was digested using T5 exonuclease according to the manufacturer’s instructions (M03363, New England Biolabs, MA, USA). As a control, pAV2 was linearized prior to T5 exonuclease treatment with *NdeI* (R0111S, New England Biolabs, MA, USA). Circular intermediates were amplified by RCA using Phi29 DNA polymerase (GE25-6400-10 Templiphi, Cytiva, MA, USA) according to the manufacturer’s protocol. Following RCA, samples were digested with *Hin*dIII (R0104S, New England Biolabs, MA, USA) or left untreated prior to sequencing or Southern blotting.

### Sequencing

Extrachromosomal DNA was extracted from infected cells using the Hirt protocol (39) and prepared for Oxford Nanopore sequencing using the ligation sequencing kit (LSK109, Oxford Nanopore Technologies, Oxford, UK) and the native barcoding expansion kit (EXP-NBD104, Oxford Nanopore Technologies, Oxford, UK) according to the manufacturers’ protocols. The samples were sequenced using a MinION sequencing device and flow cell (FLO-MIN_106, Oxford Nanopore Technologies, Oxford, UK). For PacBio sequencing, the Hirt DNA was first sheared and size selected for fragments >5 kb using the BluePippin device (Sage Science, MA, USA). Then, libraries were prepared (100-938-900, SMRTbell express template prep kit 2.0, PacBio, CA, USA) and sequenced on the PacBio Sequel II (PacBio, CA, USA) at the Functional Genomics Center (FGCZ, Zurich, Switzerland).

### Data analysis

Data analysis was performed as previously described in reference (38). Briefly, fastq-format files were transformed to fasta-format and subjected to blastn analysis against the AAV2 (Genbank #NC_001401) or rAAV genome sequences. Hits with *e*-values > 0.1 were removed, and the sum of the remaining hits was calculated for every read. Dot plots were drawn for reads with a sum of hits >3,000. The dot plots were categorized manually. The code for the bioinformatics analysis can be found in Meier et al. (38).

## Data Availability

The raw sequencing data has been deposited at the National Center for Biotechnology Information Sequence Read Archive (NCBI SRA) under accession number: PRJNA1037298. .

## SUPPLEMENTAL MATERIAL

Supplemental material jvi.01282-24-s0001.docx Figures S1 to S6.

ASM does not own the copyrights to Supplemental Material that may be linked to, or accessed through, an article. The authors have granted ASM a non-exclusive, world-wide license to publish the Supplemental Material files. Please contact the corresponding author directly for reuse.
