# Supplementary material for: AAV2 can replicate its DNA by a rolling hairpin or rolling circle mechanism, depending on the helper virus
Source: J Virol. 2024 Oct 9;98(11):e01282-24. doi: 10.1128/jvi.01282-24 (PMC11575299; doi:10.1128/jvi.01282-24)
Supplement: Supplemental material — Figures S1 to S6. [file jvi.01282-24-s0001.docx]

**Supplementary Data**

**Fig. S1. Read and Southern Blot analysis of AAV2 DNA replication intermediates in different cell types.**

Southern blot of untreated or *Hin*dIII treated Hirt DNA extracted at 24 or 48 hpi from (A) Vero, (B) HeLa or (C) BJ cells mock infected, infected with AAV2, or co-infected with AAV2 and either HSV-1 (MOI 0.1 or 1) or AdV5 (MOI 0.1 or 1). Bands were visualized with a *rep*-specific probe. (D) Read analysis of Hirt DNA extracted at 48 hpi from BJ and HeLa cells co-infected with AAV2 and either HSV-1 (MOI 0.1) or AdV5 (MOI 0.1). Table shows ratios of the different categories of AAV2 DNA replication products in BJ (56 reads) and HeLa cells (278 reads).

**Fig. S2. Dot plots of replication intermediates used for MSA analysis.**

Dot plots of the six (A) HT and (B) alternating reads used for MSA analysis.

**Fig. S3. Schematic representation of the HSV KOS-37 BAC manipulations by recombineering.** (A) Linear scheme of the circular KOS-37 BAC. (B) Magnified regions targeted by manipulations. (C) For deletion of ICP27, the galK expression cassette from pGalK (Warming et al., 2005) was amplified by PCR with the primers for_galK_d27 (5’-tgg cgc ttc act acg agc agg aga tcc aga ggc gcc tgt ttg atg tat gac ctg ttg aca atta at cat cgg ca-3’) and rev_galK_d27 (5’-aag gac aac acg tgg ggc gat ttg ttt gaa atg ttt tgt ttt tat tgt act cag cac tgt cct gct cct t-3’), and electroporated into *E.coli* SW102 harboring the HSV-1 KOS bacterial artificial chromosome (BAC). Electroporated bacteria were selected on M9 minimal plates containing chloramphenicol (CAM) and galactose. Bacteria showing successful recombineering were induced and prepared as electrocompetent cells. Electroporation with double stranded (ds) 100bp oligos (ICP27_flank: 5’-tgg cgc ttg act acg agc agg aga tcc aga ggc gcc tgt ttg atg tat gag tac aat aaa aac aaa aca ttt caa aca aat cgc ccc acg tgt tgt cct t-3’) were introduced into the bacteria and selected on M9 minimal plates containing CAM and 2-deoxy-galactose (DOG). The resulting BAC was designated fKOS_∆27. (D) Following ICP27 deletion and to insert the rep_cap9 cassette the galK expression cassette was amplified by PCR using the primers for_Hind3_galK (5’-tat aag ctt cct gtt gac aat taa tca tcg gca-3’) and rev_Hind3_galK (5’—gtg aag ctt cag cac tgt cct gct cct t-3’) with pGalK (Warming et al., 2005) as template. The amplimer was cut on both sides with *Hin*dIII and cloned into a plasmid which contains the *rep* and *cap* sequences of AAV9 and was synthesized at ATUM (Newark, CA). From this plasmid, a fragment containing the rep-cap9 and galK cassettes as well as flanking sequences homologous to the HSV-1 thymidine kinase gene was excised and electroporated into *E.coli* SW102 cells carrying fKos_∆27. Recombinant bacteria were selected for galK+ as described above. Finally, the galK cassette, situated within the AAV rep_cap9 cassette was deleted by recombineering with a ds 100 bp oligo (5’-caa acg ggt gcg cga gtc agt tgc gca gcc atc gac gtc aga cgc gga agc ttc gat ca acta cgc aga cag gta cca aaa caa atg ttc tcg tca cgt g-3’). The integrity of the manipulated region was confirmed by PCR amplification and sequencing. The resulting BAC was designated fKOS ∆27 ∆TK rep/cap. To reconstitute recombinant HSV isolated HSV-1 BAC DNA fKOS∆27∆TK rep/cap was treated with Cre-recombinase (M0298SNEB, Ipswich, MA, USA) for 30 min at 30°C. Subsequently, the BAC DNA was transfected into Vero 2-2 cells. Progeny virus was harvested and used for infection of fresh cells. The Cre-mediated recombination was confirmed by visualization of absent GFP expression. The resulting recombinant HSV-1 vectors were designated rHSV-1 rep/cap (HSV-1RC).

Reference:

Warming S, Costantino N, Court DL, Jenkins NA, Copeland NG. 2005. Simple and highly efficient BAC recombineering using galK selection. Nucleic Acids Res 33:1–12. https://doi.org/10.1093/nar/gni035

**Fig. S4. Dot plots showing two mixed reads from Oxford Nanopore sequencing.**

Sequencing read analysis of Hirt DNA from cells co-infected with AAV2, rAAVGFP and AdV5. Two dot plots from two reads that show recombination of a total of 6399 reads are illustrated. Recombination ratio amounts to 0.03%. The sequence indicated in grey is of unknown origin.

**Fig. S5. Relative viral load over time.**

qPCR analysis of lysates of infected cells. Cells were mock infected, infected with AAV2 (MOI 100), HSV-1 (MOI 0.1 or 1) or AdV5 (MOI 0.1 or 1) or co-infected with AAV2 (MOI 100) and either HSV-1 (MOI 0.1 or 1) or AdV5 (MOI 0.1 or 1). Cells were harvested at 24 and 48 hpi and DNA was isolated. Viral DNA was detected using specific primers for AAV2 Rep helicase domain (fwd: 5’-ATT GAC GGG AAC TCA ACG AC -3’, rev: 5’-ATT CAT GCT CCA CCT CAA CC-3’), HSV-1 genomic ICP0 (6) and AdV5 E1A (7). Relative viral load of (A) AAV2, (B) AdV5, and (C) HSV-1 were calculated and normalized to GAPDH (8). Graphs show log-transformed viral load normalized to GAPDH. Significance was calculated using one-way ANOVA (ns P > 0.5).

**Fig. S6. AAV2 Rep and VP1/2/3 protein expression.**

Western blot analysis of infected cell lysates. Cells were mock infected, infected with AAV2 (MOI 100) or co-infected with AAV2 and either HSV-1 (MOI 0.1 or 1) or AdV5 (MOI 0.1 or 1). Cells were harvested at 24 and 48 hpi. Total protein was separated by SDS-PAGE and blotted onto nitrocellulose membrane. Specific proteins were detected with the indicated antibodies with GAPDH as loading control. Protein levels of (A) AAV2, (B) Rep, and (C) VP1/2/3 were quantified using Fiji. Graphs show log-transformed signal normalized to GAPDH. Significance was calculated using one-way ANOVA (ns P > 0.07, * P < 0.02, *** P < 0.0008, **** P < 0.0001).
